# The relationship between academic stress and depression among college students during the COVID-19 pandemic: a cross-sectional study from China

**DOI:** 10.1186/s12888-024-05506-8

**Published:** 2024-01-12

**Authors:** Baoling Chen, Weiwei Wang, Shanlin Yang

**Affiliations:** 1 School of Management, Hefei University of Technology, 193 Tunxi Rd, Hefei, Anhui 230009 People’s Republic of China; 2 School of Finance and Public Administration, Anhui University of Finance & Economics, #962 Caoshan Road, Bengbu City, Anhui China; 3 Internal Medicine Department, The Third People’s Hospital of Bengbu, No. 38, Shengli Middle Road, Bengbu City, Anhui People’s Republic of China

**Keywords:** Academic stress, Coping style, COVID-19, Interpersonal relations, Sleep quality, Depression

## Abstract

**Background:**

The COVID-19 pandemic is a global public health crisis. During the COVID-19 pandemic, mental health has attracted great attention. However, there is a lack of research on the relationship between academic stress and depression in Chinese college students and its mechanisms. Therefore, this study investigated the mechanisms of coping style, sleep quality, and interpersonal relationship in academic stress and depression among college students.

**Methods:**

The cross-sectional study was conducted from May to June 2022 through face-to-face questionnaires with college students in Anhui Province, China. The questionnaires included sociodemographic information, the Simplified Coping Style Questionnaire, and the Self-Rating Depression Scale. Ordered logistic regression model was used to study the relationship between academic stress and depression of college students during the COVID-19 pandemic through the mechanism analysis of coping style, sleep quality and interpersonal relationship.

**Results:**

Two thousand thirty-three Chinese college students participated in the study, including 1,285 female and 748 male college students, with an average age 19.81 years old (*SD* = 1.22 years old). The results showed that (1) Academic stress had a significant impact on depression in college students under the background of COVID-19 (*p* < 0.01); (2) The influence of academic stress on depression had a difference in work experience as student cadres, which showed that college students who had served as student cadres were less affected by academic stress (*p* < 0.10), college students who had not served as student cadres were greatly affected by the academic stress (*p* < 0.05); (3) College students’ attitudes toward COVID-19 significantly affected depression (*p* < 0.01); (4) Counselors’ concern had a significant impact on college students’ depression (*p* < 0.01); (5) Positive coping style, high quality sleep and good interpersonal relationship were the important mechanisms of the impact of academic stress on college students’ depression.

**Conclusions:**

This study provides new findings for in-depth understanding of the relationship between academic stress and depression among college students in China during the COVID-19 pandemic, which is conducive to the provision of targeted intervention measures for the mental health of college students.

## Introduction

The COVID-19 pandemic is a global public health crisis. The COVID-19 pandemic has a significant impact on depression, anxiety and mental distress [1]. There are more than 70 million new cases of depression, 90 million new cases of anxiety, and hundreds of millions of new cases of sleep disorders [2]. The prevalence of depression in college students increases significantly during the period of COVID-19 [3], and the incidence of depression increases year by year [4]. According to the “China National Mental Health Development Report (2021–2022)”, the detection rate of depression risk in the 18–24 age group is as high as 24.1%, significantly higher than other age groups [5]. The healthy growth of college students is related to the great rejuvenation of the Chinese nation. Therefore, it is of great significance to pay attention to the mental health of college students and explore the depression and its influencing mechanisms.

Academic stress is an important factor affecting college students’ depression [6]. Academic stress is used to describe the stress an individual may experience in the educational environment and in relation to a variety of academic related issues [7]. According to traditional Confucianism, Chinese education tends to focus mainly on various exams or tests, and schools and parents one-sided pursuit of high scores, leading to great pressure on Chinese students [8, 9]. The COVID-19 pandemic increases the academic stress, possibly due to the physical distancing measures in response to COVID-19. Colleges have turned to emergency forms of online learning, thus further exacerbating the academic stress on students, who may experience reduced motivation to learn and increased pressure to study independently [10]. In addition, COVID-19 exposes college students to new stressors, such as social isolation and worries about loved ones [11], which can be frustrating and lead to mental health problems [12]. Depression is positively influenced by long-term study stress, especially in the context of the COVID-19 pandemic [13]. Research shows that during the pandemic, students with irregular diets and work and rest are more likely to have stress, depression and anxiety, and the depression rate of college students is 35.12%, higher than that of college students with anxiety [14]. Therefore, further research on academic stress and mental health, especially academic stress and depression, is of great importance.

In addition, the impact of academic stress on depression may be influenced by multiple factors, such as coping style, sleep quality, and interpersonal relationship [15–17]. Coping style is the method, means or strategy used to deal with internal and external environmental requirements and related emotional distress [18]. Research has found that strengthening resilience and coping strategies in the face of the COVID-19 pandemic could have an important impact on mental health [19]. A variety of coping strategies, such as behavioral activation and mindfulness practices, can reduce stress and promote resilience and recovery, thereby promoting mental health [20]. Sleep quality is another factor. Academic stress has a significant negative impact on college students’ sleep quality [21], while sleep quality is significantly associated with depression [22]. Related studies have found that sleep quality plays an important role in predicting both physical and mental health [23]. Students with poor sleep quality are 3.28 times more likely to develop depressive symptoms [24]. Another factor is interpersonal factors, such as quarantine and social isolation, which are considered to be high risk factors for depression [25, 26]. In a diverse sample of college students, concerns about COVID-19 infection and loneliness have been found to be associated with worse mental health among students of color [27]. Moreover, high interpersonal sensitivity can aggravate depression [28].

Through the above research, the outbreak of COVID-19 has increased the academic stress, and college students have a high level of depression [29]. Although some studies have suggested that coping style, sleep quality, and interpersonal relationship are associated with depression, the exact mechanisms are not clear. The ecological model of the stress process provides us with a theoretical paradigm for studying the combined effects of environment and social psychology on college students’ depression [30]. This model reminds people to pay attention to both the ecological system and the stress variables generated in it [31]. This model can reasonably explain the mechanisms of academic stress and depression. On the basis, individual micro-level depression can be regarded as the result of the influence of macro structural variables through the meso-level variables (academic stress). Based on this theory, this study explored the impact of academic stress on depression and the underlying mechanisms of coping style, sleep quality, and interpersonal relationship in this relationship. In addition, relevant intervention measures were proposed in combination with traditional Chinese Confucianism.

The overall objective of this study was to examine the relationship between academic stress and depression of college students in China during the COVID-19 pandemic, and to explore the underlying mechanisms. Based on the data from the research group of studying the influence of COVID-19 on mental health of college students, this study was conducted using the Ologit model. The following specific objectives were proposed around the overall objective. Firstly, to examine whether academic stress had an impact on depression. Secondly, to examine whether the impact of academic stress on depression had a difference in work experience as student cadres. Finally, the mechanisms of coping style, sleep quality and interpersonal relationship were analyzed to provide insight into the transmission mechanisms. The main contributions of this study were to investigate the differences in depression between college students who were student cadres and those who were not. Secondly, this study analyzed the mechanisms between academic stress and college students’ depression during COVID-19.

## Materials and methods

### Sampling and sample size

A cross-sectional study was conducted from May to June 2022 at a university in Anhui Province, China. Anhui Province, located in central and eastern China, is an important birthplace of Chinese civilization and a major education province, with students from all over the country. The sample size was calculated by single population proportion formula using the following assumptions. The population proportion was selected as 50%, 95% confidence interval (1.96), 3% of margin error (0.03), and a design effect of 1.5. The design formula is as follows:

$${n_1} = \frac{{{z^2}P(1 - P)}}{{{e^2}}}.$$

Plugging the value n = (1.96)^2^ * 0.5(1 − 0.5)/0.03^2^ = 1067. Using cluster sampling, the sample size was multiplied by a design effect of 1.5 to satisfy intra-cluster variability [32]. Therefore, the final minimum sample size calculated was 1.5 * 1067 = 1600.

### Data collection

Before the formal investigation, a small sample of 60 college students was pre-investigated, and the questionnaires were improved according to the reliability and validity test results of the pre-investigation and feedback of the participants. At the same time, we invited psychology teachers, professional psychological counselors to evaluate the questionnaires to ensure that the content of the questionnaires would not bring unhealthy effects to the participants. Students with mental disorders such as depression were excluded. Inclusion criteria were undergraduate students who voluntarily completed the questionnaires. Graduate students and undergraduate students who did not provide informed consent were excluded. Intentions were clearly stated, informed consents were obtained, and participants were assured of privacy and confidentiality and were clearly informed that there were no right or wrong answers. This study was approved by the research ethics committee and was conducted in accordance with the Declaration of Helsinki.

In the face-to-face questionnaires, all participants answered the questionnaires, and a total of 2084 questionnaires were collected. Excluding random answers and contradictory answers, 2033 questionnaires were finally valid, with an effective recovery rate of 97.55%. Among them, 1285 (63.21%) were female college students and 748 (36.79%) were male college students, including 698 freshmen, 712 sophomore, 610 junior and 13 senior students. There were 651 (32.02%) only child and 1,382 (67.98%) not only child. The average age of the students was 19.81 years old (*SD* = 1.22 years old).

### Measures

#### Measurement of depression

The depression was measured by Self-Rating Depression Scale (SDS) developed by Zung [33]. Specifically, it contains 20 items, including 10 reverse scoring questions, and is scored on a 4-point scale. This scale is often used in the psychological self-rating depression scale. According to the results of the Chinese norm, the cut-off value of the SDS is 53 points, among which a score of 53–62 is classified as mild depression, a score of 63–72 is classified as moderate depression, and a score of 73 or above is classified as severe depression. At present, SDS is considered as a reliable depression screening tool in the Chinese population, which has been verified and widely used by previous research on Chinese college students [34]. In this study, AVE = 0.54, CR = 0.90. Confirmatory factor analysis showed that the scale provided a good fit: *χ*^2^/df = 4.93, RMSEA = 0.04, CFI = 0.99, TLI = 0.98, SRMR = 0.01. The Cronbach’s α coefficient of the scale was 0.87.

#### Measurement of academic stress

The core explanatory variable is academic stress. Academic stress is used as a continuous variable in this study [35]. Based on college students’ subjective feelings of academic stress during the pandemic, the academic stress is measured by the question “feeling academic stress” in the questionnaire. This question is rated on four levels from “no stress” to “very stressful”. The higher the score, the greater the stress.

#### Measurement of coping style

The relationship between coping style and mental health has become an important part of psychological research. The Simplified Coping Style Questionnaire (SCSQ) compiled by Xie Yaning in 1998 is used in the study. The SCSQ is compiled by combining the characteristics of Chinese people, consisting of two dimensions, positive coping and negative coping, including 20 items [36]. The positive coping dimension is composed of items 1–12, which mainly reflect the characteristics of positive coping. The negative coping dimension consists of items 13–20, which mainly reflect the characteristics of negative coping. The SCSQ adopts a four-point scale, that is, 0 means “not taking”, 1 means “occasionally taking”, 2 means “sometimes taking” and 3 means “often taking”. The reliability and validity of the SCSQ are 0.94 and 0.96, respectively, with very good reliability and validity. The population test has shown that the SCSQ reflects the characteristics of different coping styles and the relationship between coping styles and mental health. High positive coping scores are associated with low scores for psychological problems or symptoms, while high negative coping scores are associated with high scores for psychological problems or symptoms. This study posits that coping style is the mediating variable. Coping style is dummy variable, in which positive coping style is 1 and negative coping style is 0. In this study, AVE = 0.48, CR = 0.94. Confirmatory factor analysis showed that the scale provided an acceptable fit: *χ*^2^/df = 7.30, RMSEA = 0.06, CFI = 0.97, TLI = 0.96, SRMR = 0.03. The Cronbach’s α coefficient of the scale was 0.94.

#### Measurement of sleep quality

In this study, sleep quality is assumed to be the mediating variable. Sleep quality during the pandemic is evaluated by the question “How is your sleep quality” based on the subjective perception of college students. It is scored on a five-point scale from “very bad” to “very good”, and the higher the score, the better the sleep quality.

#### Measurement of interpersonal relationship

In this study, interpersonal relationship is assumed to be the mediating variable. Interpersonal relationship is evaluated through the subjective perception of college students by the question “How is your interpersonal relationship”. It is scored on a five-point scale from “very bad” to “very good”, the higher the score, the better the interpersonal relationship.

#### Measurement of control variables

There may be some confounding variables in the relationship between academic stress and depression, which need to be controlled. According to previous studies, these control variables mainly include gender, marital status, personality, alcohol drinking, physical condition, total household income, household economy decline, father’s anxiety about COVID-19, mother’s anxiety about COVID-19, negative information received about COVID-19, attitude toward the pandemic, dietary, panic, Internet dependence, counselor’s concern, tutor’s concern. Firstly, in terms of demographic characteristics, gender is a dummy variable, 1 for men and 0 for women; marital status, 1 means single, 0 means partnership or married. Personality is a categorical variable, 1 means extroverted, 2 means ambivert and 3 means introverted. Whether drinking alcohol or not is the dummy variable, drinking alcohol is 1, and not drinking alcohol is 0. Physical condition is a continuous variable, ranging from “very unhealthy” to “very healthy” on a five-point scale. Secondly, for family aspect, the total household income is classified, and “1 means less than 20,000 RMB yuan (1US dollar ≈ 6.91 RMB yuan in the year 2023); 2 means 20,000–49,999 RMB yuan; 3 means 50,000–99,999 RMB yuan; 4 means 100,000–199,999 RMB yuan; 5 means 200,000 RMB yuan or above”; Whether the household economy has declined significantly is a dummy variable, 1 is yes, 0 is no. Thirdly, in terms of the impact of COVID-19 on individuals,” the amount of negative information received about COVID-19” is rated from “very little” to “very much” on a five-point scale, with the larger the number, the more concerned an individual is about the COVID-19. The dietary during the pandemic is rated on a five-point scale, from “very bad” to “very good”, the larger the number, the better the dietary. The level of panic, from “not at all” to “very panicked”, is a four-point scale, with the greater the value, the more fearful the individual is. Internet dependence, from “not at all” to “very dependent,” is rated on a five-point scale, with the greater the value, the stronger the individual’s dependence on the Internet. Finally, for the teacher’s concern for college students, the counselor’s concern and tutor’s concern are graded on a five-point scale from “not at all” to “very concerned”, the higher the value, the more concerned the counselor and tutor are about the college students.

### Data analysis

Stata 17.0 software was used for analysis. Categorical variables were presented as frequency and percentage distributions, and continuous variables were presented as means ± standard deviations (*SD*). Descriptive statistical analyses were performed to study the demographic and other selected characteristics of the participants. Regression assumptions, such as normality tests and multicollinearity, were tested before regression analysis. Then, after controlling for demographic characteristics, family characteristics and other characteristics, Ologit model was used to explore the effect of academic stress on depression. Heterogeneity analysis was also conducted among different groups of college students, and based on the mediating effect, the effects of coping style, sleep quality and interpersonal relationship between academic stress and depression were examined.

## Results

### Descriptive statistics

Descriptive statistical analysis (Table 1) showed that the average score of depression of Chinese college students during the COVID-19 pandemic was 1.97 (*SD* = 0.91), indicating that most of them were mildly depressed and their depression was still under control. The average academic stress of college students was 2.78 (*SD* = 0.74), indicating that most of them felt stressed during the COVID-19 pandemic. College students are the main body of higher education. Most colleges have set up the course of Mental Health Education, which teaches pressure regulation and emotion management. It could be seen from the questionnaires that most college students could actively cope with pressure, reaching 90.56%. In terms of sleep quality, most of the students said that their sleep quality was at a normal level, except for a few students who liked to stay up late, which was inseparable from psychological quality. In terms of interpersonal relationship, most students said they were in the “average and good” range. In addition, 36.79% of the sample college students were male, 76.34% were single, 60.11% were ambivert, most of them had served as student cadres, and only 14.36% drank alcohol. The physical condition of college students was generally good, 79.44% of them were in “relatively healthy or very healthy”. The average total household income last year was 2.93 (*SD* = 1.23), the minimum value was 1, the maximum value was 5, and the mean value was close to 3, indicating that most of the family income of college students was between 50,000 and 99,999 RMB yuan. 58.73% of college students said the COVID-19 pandemic had caused a decline in the household economy. The majority of parents’ anxiety during the COVID-19 pandemic was occasionally and often. College students’ attitude towards the pandemic was relatively concerned and very concerned, indicating that college students were very concerned about the COVID-19 pandemic, and would consider whether the pandemic would bring difficulties and obstacles to their study, life and employment choices, thus bringing certain psychological pressure. Despite the positive improvement in Chinese pandemic prevention and control situation, college students still received more negative information about the COVID-19. 18.49% of the students had quarantine experience. During the period of closed campus management, college students said their diet was average and good. College students expressed a certain level of panic during the pandemic, with an average of 1.73 (*SD* = 0.54). The Internet dependence of college students was more serious. Only 2.21% of them were not dependent at all, while 59.32% of them were relatively dependent or very dependent.

**Table 1:** Definitions and descriptive statistics for each type of variable

Variable		Definition	Mean	SD	Min	Max
Dependent variable	Depression	1 = no; 2 = mild; 3 = moderate; 4 = severe	1.971	0.913	1	4
Independent variable	Academic stress	1 = no stress, 4 = very stressful	2.783	0.742	1	4
Mediating variable	Coping style	0 = negative, 1 = positive	0.906	0.293	0	1
	Sleep quality	1 = very bad, 5 = very good	3.331	0.953	1	5
	Interpersonal relationship	1 = very bad, 5 = very good	3.509	0.730	1	5
Personal characteristics	Gender	0 = women, 1 = men	0.368	0.482	0	1
	Marital status	0 = non-single, 1 = single	0.763	0.425	0	1
	Personality	1 = extrovert; 2 = ambivert; 3 = introvert	2.013	0.632	1	3
	Student cadre	0 = no, 1 = yes	0.625	0.484	0	1
	Alcohol drinking	0 = no, 1 = yes	0.144	0.351	0	1
	Physical condition	1 = very unhealthy, 5 = very healthy	4.038	0.774	1	5
Family characteristics	Total household income (RMB yuan)	1 = less than 20,000; 2 = 20,000–49,999; 3 = 50,000–99,999; 4 = 100,000–199,999; 5 = 200,000 or above	2.933	1.234	1	5
	Household economy decline	0 = no, 1 = yes	0.587	0.492	0	1
	Father’s anxiety	1 = no, 4 = very anxious	2.157	0.726	1	4
	Mother’s anxiety	1 = no, 4 = very anxious	2.192	0.723	1	4
Impact of the pandemic on individuals	Attitude towards the pandemic	1 = not at all, 5 = very concerned	3.528	0.892	1	5
	Negative information received	1 = very little, 5 = very much	3.201	0.907	1	5
	Quarantine experience	0 = no, 1 = yes	0.185	0.388	0	1
	Dietary	1 = very bad, 5 = very good	3.387	0.882	1	5
	Level of panic	1 = not at all 4 = very panicked	1.731	0.542	1	4
	Internet dependence	1 = not at all 4 = very dependent	3.585	0.889	1	5
Teacher’s concern	Counselor’s concern	1 = not at all, 5 = very concerned	4.068	0.910	1	5
	Tutor’s concern	1 = not at all, 5 = very concerned	2.463	1.148	1	5

### Regression results of the Ologit model

The dependent variable in this study was the depression, which ranged from 1 to 4. It was a discrete and ordered variable. Therefore, the Ologit model was constructed for regression. Table 2 reported the estimated results by using the Ologit model. Model 1 was the result of the influence of academic stress on the depression of college students under the COVID-19 pandemic without including control variables. The analysis results showed that academic stress positively affected the depression of college students at the significance level of 1%, indicating that the greater the academic stress, the more prone to depression.

**Table 2:** Ologit model regression results of the influence of academic stress on college students’ depression during the COVID-19 pandemic

Variable	Model (1)	Model (2)	Model (3)	Model (4)	Model (5)
	Depression	Depression	Depression	Depression	Depression
Academic stress	0.405^***^ (0.057)	0.352^***^ (0.058)	0.286^***^ (0.059)	0.164^***^ (0.062)	0.166^***^ (0.062)
Gender		0.376^***^ (0.090)	0.420^***^ (0.091)	0.463^***^ (0.095)	0.470^***^ (0.095)
Marital status		−0.295^***^ (0.101)	−0.268^***^ (0.101)	−0.206^**^ (0.103)	−0.196^*^ (0.103)
Personality		0.221^***^ (0.071)	0.219^***^ (0.071)	0.210^***^ (0.073)	0.212^***^ (0.073)
Student cadre		−0.151^*^ (0.090)	−0.145 (0.090)	−0.140 (0.092)	−0.141 (0.092)
Alcohol drinking		0.416^***^ (0.124)	0.451^***^ (0.124)	0.362^***^ (0.127)	0.385^***^ (0.127)
Physical condition		−0.471^***^ (0.057)	−0.456^***^ (0.057)	−0.271^***^ (0.060)	−0.261^***^ (0.061)
Total household income			−0.078^**^ (0.036)	−0.067^*^ (0.037)	−0.068^*^ (0.037)
Household economy decline			−0.065 (0.094)	−0.137 (0.097)	−0.140 (0.097)
Father’s anxiety			0.198^**^ (0.084)	0.142^*^ (0.086)	0.145^*^ (0.086)
Mother’s anxiety			0.145^*^ (0.084)	0.070 (0.086)	0.071 (0.087)
Attitude toward the pandemic				−0.197^***^ (0.051)	−0.185^***^ (0.051)
Negative information received				0.260^***^ (0.050)	0.251^***^ (0.051)
Quarantine experience				−0.130 (0.111)	−0.115 (0.111)
Dietary				−0.581^***^ (0.055)	−0.566^***^ (0.055)
Level of panic				0.379^***^ (0.085)	0.380^***^ (0.085)
Internet dependence				0.145^***^ (0.051)	0.141^***^ (0.051)
Counselor’s concern					−0.187^***^ (0.057)
Tutor’s concern					−0.052 (0.045)
LR statistics	52.14	196.31	230.63	450.65	461.90
Pseudo R^2^	0.011	0.042	0.049	0.095	0.098
N	2033	2033	2033	2033	2033

*Note*: (1) *p < 0.10, **p < 0.05, ***p < 0.01. (2) The values in parentheses are standard errors

Model 2 was the effect of academic stress on depression of college students after the inclusion of demographic control variables, which was consistent with model 1. Gender positively affected the depression of college students at the significance level of 1%, indicating that male college students had more serious depression than female college students. Marital status negatively affected the depression of college students at the significance level of 1%, indicating that single students were less likely to be depressed than non-single students. The possible reason was that students who were in love or married had fewer opportunities to meet each other due to the pandemic control measures, resulting in urgent yearning, worries and other emotions, and were more likely to be depressed than single students. Personality positively affected the depression of college students at the significance level of 1%, which was consistent with most studies [37, 38]. Alcohol drinking positively affected depression of college students at the significance level of 1%. The more college students like drinking alcohol, the more prone to depression. The physical condition of college students negatively affected the depression at the significance level of 1%, indicating that the healthier college students were, the less likely they were to be depressed.

Model 3 was the effect of academic stress on depression of college students after further incorporating family characteristic control variables, which was consistent with model 1. The total household income negatively affected the depression of college students at the significance level of 5%, which indicated that compared with students from poor families, college students from rich families were less prone to depression. This may be due to the economic support of their families, college students had relatively less pressure on life and employment. During the pandemic, the anxiety of fathers positively affected college students’ depression at the significance level of 5%, while the anxiety of mothers positively affected college students’ depression at the significance level of 10%, which indicated that parents’ emotions could also affect college students, especially fathers. Because in China, fathers are often the backbone of the family, the main source of the family economy and spiritual support.

Model 4 showed the effect of academic stress on depression of college students after adding the control variables of the impact of COVID-19 on individuals, which was consistent with model 1. During the pandemic, college students’ attitudes towards the pandemic, the amount of negative information they received, their diet and their dependence on the Internet all affected the depression at a significant level of 1%, indicating that the more college students paid attention to the pandemic, the less likely they were to be depressed. It may be that the more they know about the pandemic, the more rational they can view it. The more negative information received about the pandemic, the more college students were prone to depression. The better the diet, the less college students were prone to depression. The higher the level of panic and the more dependent on the Internet, the more college students were prone to depression.

Model 5 showed the effect of academic stress on depression of college students after adding the control variables of the influence of college on individuals, which was consistent with Model 1. The care of counselors significantly affected the mental health of college students at the 1% significance level, which benefited from the advantages of the Chinese college counselor system. In college, the counselor is the “closest person” to the students and becomes the first choice for college students when they encounter difficulties. The concern of tutors did not significantly affect the mental health of college students. This is because in the undergraduate stage, tutors mainly guide dissertations at the time of graduation, and rarely interact with students in ordinary times, especially in life. This further demonstrates the remarkable effect of the professional construction of the counselor team with Chinese characteristics.

### Heterogeneity analysis

The mental health of college students can be affected by individual psychological resilience, and serving as a student cadre is the key to enhance the psychological resilience of college students. The question thus raised is whether there will be differences in the relationship between academic stress and mental health in those who have served as student cadres or not. Therefore, the sample was distinguished by whether they had been a student cadre to investigate the effect of academic stress on the mental health of college students.

**Table 3:** Analysis of heterogeneity

Variable	Depression	Depression
	Served as a student cadre	Never served as a student cadre
Academic stress	0.147^*^	0.235^**^
	(0.079)	(0.104)
Control variable	Control	Control
LR statistics	294.58	188.75
Pseudo R^2^	0.102	0.104
N	1,271	762

*Note*: (1) *p < 0.10, **p < 0.05, ***p < 0.01. (2) The values in parentheses are standard errors

The sub-sample test results in Table 3 showed that academic stress had a significant impact on college students who had served as student cadres or not. Among those who had served as student cadres, the influence of academic stress on psychological depression was less, with a coefficient of 0.147, which was significant at the level of 10%. For those who had not served as student cadres, the influence of academic stress on psychological depression was greater, with a coefficient of 0.235, which was significant at the significance level of 5%. Students who can serve as student cadres in colleges are often excellent and have strong communication skills. Through the tempering of student cadres, students’ psychological qualities are further exercised. Therefore, students who have served as student cadres can reduce the degree of depression more from academic stress.

### Robustness analysis

In order to test whether the regression results of academic stress on college students’ depression were stable, we used two methods to do a robustness test. The first method considered the construction of ordered probit model to verify the credibility of the research results, and the results were shown in Table 4. The second was to replace the original core explained variable with the degree of self-assessment depression, and test whether the regression result was significant. The questionnaire “How depressed do you think you are” was used to assess the self-assessment depression of college students. It was a subjective question, ranging from “not at all” to “very depressed” on a five-point scale. The higher the value, the more depressed college students were. The results are shown in Table 5.

**Table 4:** Oprobit model regression results of the influence of academic stress on college students’ depression during the COVID-19 pandemic

Variable	Model (1)	Model (2)
Academic stress	0.247^***^ (0.034)	0.100^***^ (0.037)
Control variable	Uncontrol	Control
LR statistics	54.43	462.13
Pseudo R^2^	0.012	0.098
N	2033	2033

*Note*: (1) *p < 0.10, **p < 0.05, ***p < 0.01. (2) The values in parentheses are standard errors

**Table 5:** Ologit model regression results of the influence of academic stress on college students’ self-assessment depression during the COVID-19 pandemic

Variable	Model (1)	Model (2)
	Self-assessment depression	Self-assessment depression
Academic stress	0.568^***^ (0.060)	0.339^***^ (0.066)
Control variable	Uncontrol	Control
LR statistics	92.01	403.00
Pseudo R^2^	0.020	0.088
N	2033	2033

*Note*: (1) *p < 0.10, **p < 0.05, ***p < 0.01. (2) The values in parentheses are standard errors

The results in Tables 4 and 5 were consistent with the regression results in Table 2 above. In Model 1 and Model 2 of Tables 2 and 4, and 5, Model 1 was the result without adding control variables, while Model 2 was the result with adding control variables. It could be seen that the results of Model 1 and Model 2 were consistent. In addition, in Tables 2 and 4, academic stress had a significant effect on the depression of college students at the significance level of 1% without adding control variables. After the addition of control variables, the academic stress on the depression of college students was still significant at the significance level of 1%, which showed the robustness of the results. In Table 5, academic stress affected the self-assessment depression, regardless of whether control variables were added, the results were at the 1% significance level. The significance results of other variables were also consistent with the results in Table 2. Through these two robustness tests, it could be seen that it was consistent with the conclusion of the basic model above, which confirmed that the results of this study were stable.

### Mechanism analysis: the mediating role of coping style, sleep quality and interpersonal relationship

This study applies for the mediation effect test method proposed by Wen et al. [39] to test the mediation effect. As can be seen from Table 6, Model 1 was the influence of academic stress on depression when all control variables were included. Academic stress positively affected the depression of college students at the significance level of 5%. Model 2 was the influence of academic stress on coping style when all control variables were included. Among them, academic stress had a negative impact on coping style at the significance level of 5%, indicating that the higher academic stress college students had, the more negative coping styles they may have. In Model 3, when all the control variables were included, academic stress and coping style variables were added at the same time, the results showed that coping style negatively affected the depression at the significance level of 1%. The influence of academic stress on depression of college students decreased from 16.6% in Model 1 to 15.5% in Model 3, indicating that coping style was one of the mechanisms of academic stress on the depression. Coping style played a mediating role in the influence of academic stress on the depression of college students. The depression of college students can be improved by adjusting their coping style.

**Table 6:** Academic stress and depression of college students: the mediating role of coping style

Variable	Model (1)	Model (2)	Model (3)
	Depression	Coping style	Depression
Academic stress	0.166^**^ (0.062)	−0.236^**^ (0.113)	0.155^**^ (0.063)
Coping style			−1.013^***^ (0.156)
Control variable	Control	Control	Control
LR statistics	460.83	99.69	505.39
Pseudo R^2^	0.098	0.078	0.107
N	2033	2033	2033

*Note*: (1) *p < 0.10, **p < 0.05, ***p < 0.01. (2) The values in parentheses are standard errors

It can be seen from Table 7. Model 1 was the impact of academic stress on college students’ depression when all control variables were included. Academic stress positively affected the depression of college students at the significance level of 5%. Model 2 was the impact of academic stress on sleep quality when all control variables were included. Among them, academic stress had a significant negative effect on sleep quality at the significance level of 1%, indicating that the higher the academic stress, the worse the sleep. In Model 3, when all the control variables were included, academic stress and sleep quality variables were added at the same time, the results showed that sleep quality negatively affected the depression at the significance level of 1%. The influence of academic stress on depression of college students decreased from 16.6% in Model 1 to 13.3% in Model 3, indicating that sleep quality was one of the mechanisms of academic stress on the depression. Sleep quality played a mediating role in the influence of academic stress on the depression of college students. The depression of college students can be improved by improving the quality of sleep.

**Table 7:** Academic stress and depression of college students: the mediating role of sleep quality

Variable	Model (1)	Model (2)	Model (3)
	Depression	Sleep quality	Depression
Academic stress	0.166^**^ (0.062)	−0.249^***^ (0.065)	0.133^**^ (0.063)
Sleep quality			−0.331^***^ (0.061)
Control variable	Control	Control	Control
LR statistics	460.83	1325.71	491.29
Pseudo R^2^	0.097	0.245	0.104
N	2033	2033	2033

*Note*: (1) *p < 0.10, **p < 0.05, ***p < 0.01. (2) The values in parentheses are standard errors

It can be seen from Table 8. Model 1 showed the influence of academic stress on the depression of college students when all control variables were included. Academic stress positively affected the depression of college students at the significance level of 5%. Model 2 was the influence of academic stress on interpersonal relationship when all control variables were included. Among them, academic stress had a negative impact on interpersonal relationship at the significance level of 5%, indicating that the higher the academic stress, the worse the interpersonal relationship. In Model 3, when all the control variables were included, academic stress and interpersonal relationship variables were added at the same time, the results showed that interpersonal relationship negatively affected the depression at the significance level of 1%. The influence of academic stress on depression of college students decreased from 16.6% in Model 1 to 14.5% in Model 3, indicating that interpersonal relationship was one of the mechanisms of academic stress on the depression of college students. Interpersonal relationship played a mediating role in the influence of academic stress on the depression of college students. The depression of college students can be improved by improving interpersonal relationship.

**Table 8:** Academic stress and depression of college students: the mediating role of interpersonal relationship

Variable	Model (1)	Model (2)	Model (3)
	Depression	Interpersonal relationship	Depression
Academic stress	0.166^**^ (0.062)	−0.182^**^ (0.066)	0.145^**^ (0.063)
Interpersonal relationship			−0.337^***^ (0.069)
Control variable	Control	Control	Control
LR statistics	460.83	612.99	486.09
Pseudo R^2^	0.097	0.141	0.103
N	2033	2033	2033

*Note*: (1) *p < 0.10, **p < 0.05, ***p < 0.01. (2) The values in parentheses are standard errors

## Discussion

The results of this study indicate that academic stress significantly affects depression among Chinese college students. This finding is consistent with the results of studies conducted in South Korea [40], Netherlands [41], and Turkey [42]. The reason for this consistency may be that the same study population (college students) was used, despite differences in the screening tools used, such as the academic stress scale or the depression scale. Mental disorders such as depression and substance abuse are more common during the COVID-19 pandemic [43], and three-quarters of college students report feeling stressed [44]. Overall, stress in college students is significantly associated with depression [45]. Distance learning, loneliness and academic stress brought about by the COVID-19 have aggravated the psychological distress of college students [46]. In addition to the heavy course load [47], they also face various competitions, family pressure. Coupled with the impact of COVID-19 on the economy, the employment competition will become more intense, which is most notably reflected in the academic competition. All these factors further aggravate the tension and worry of college students. During the pandemic, in addition to the competition for on-campus scholarships and honorary titles, it is also highlighted by the significant increase in the number of Chinese undergraduates who apply for postgraduate. To reduce the academic stress, we need to work together. Family, school and society should be combined to improve the quality of education, strengthen mental health education and improve the psychological quality of college students, so as to cope with the adverse effects of excessive academic stress on physical and mental health.

The study also finds that the influence of academic stress on depression of college students is different whether they have the experience of working as student cadres. College students who have served as student cadres are less affected by academic stress. College students who have not served as student cadres are more affected by academic stress. During the COVID-19 pandemic, there is still a lack of attention to the depression of student cadres. This is also a new finding of this study, which enriches the related research. This difference may be due to the fact that college students who have been student cadres have more self-management and other abilities exercised in universities, and they tend to have more empathy [48] and show more positive social mentality [49]. College students’ emotion management ability plays an important role during COVID-19 [50]. Entering the new era, China puts forward new requirements for talent quality [51]. Student cadres are the backbone of college students. In the process of serving as student cadres, college students continue to grow and gain recognition. While improving their cultural soft power, they also meet the psychological needs of recognition [52, 53]. The academic achievement of student cadres is significantly higher than that of non-student cadres, and their learning and cognition ability and communication ability are both higher than those of non-student cadres [54]. Therefore, colleges should provide more opportunities for student cadres to exercise, create a level playing field, enrich college life, and strive to enhance the mental toughness of college students and improve their mental health.

In addition, college students’ attitudes toward the COVID-19 and the amount of negative information received significantly affect their depression, which is consistent with the previous study [55]. Facing the potential outbreak of COVID-19 at any time, college students are often in a tense state. On the one hand, once there are infection cases in the school, or individuals become the object of close contact or secondary close contact, they will be quarantined and controlled at any time. On the other hand, the spread of COVID-19 and its unpredictable end are of great concern. Therefore, college students are very concerned about the changes of the COVID-19. All kinds of negative information on the Internet will increase the psychological burden of college students [56]. Government and colleges should strengthen pandemic prevention policies and knowledge dissemination to reduce the spread of negative information and improve college students’ understanding and awareness of pandemic prevention policies and their perception of health threats. During the COVID-19 pandemic, college counselors in China have been the main force in pandemic prevention and student affairs, playing a role in protecting the lives and health of students. It is the key to alleviating the psychological problems of college students that counselors give them spiritual care and psychological counseling in daily life. This is the advantage of the counselor system with Chinese characteristics. Colleges should also strengthen the ability and quality training of college counselors, care for this group, and constantly improve the professional identity and happiness of college counselors.

This study also used mediating effect analysis to analyze the mechanisms of academic stress and depression. Positive coping style significantly reduced the depression of college students, with an average reduction of 1.1%. During the COVID-19 pandemic, college students of different genders and grades have different coping styles. The more positive the coping style, the lower the level of depression, which is consistent with the current research [57, 58]. Positive coping can enhance psychological resilience [59] and reduce the risk of anxiety and depression. Negative coping is associated with more negative emotions and mental disorders [60]. In addition, it may also be related to the influence of Confucianism on Chinese college students from an early age. The core of Chinese traditional culture is Confucianism, which is the rich moral and spiritual wealth left by the Chinese nation. One of the core ideas of Confucianism is how to become a person, that is, to explore how to become a person worthy of social expectations. Confucianism believes that people have responsibilities in the world and that suffering is conducive to personal growth. Therefore, in the face of setbacks and suffering, there is no need to fear and escape, but to face them bravely and to improve oneself in the process of setbacks and suffering, emphasizing the role of positive coping in stress. Therefore, colleges should make efforts to cultivate the correct ideological cognition of college students, improve their health literacy, and guide them to adopt more positive coping to avoid the generation of psychological problems. College students can enhance their self-confidence by smiling in front of the mirror every day, facing difficulties and challenges with positive attitudes, and making friends with enterprising people to promote their own optimistic mood.

The study found that high quality sleep significantly reduced depression of college students, by an average of 3.3%. This is consistent with the findings of a large-scale survey during the COVID-19 pandemic [61]. On the other hand, a study of college students in the US finds that although college students go to bed later than before the pandemic, the amount of sleep increase, so the impact of COVID-19 on stress and sleep may not be entirely negative [62]. However, our findings are consistent with more research findings, especially for the research of Chinese college students. Some studies have found that college students have high rates of sleep problems and high perceived stress during the COVID-19 pandemic [63]. Some scholars have found through large-scale surveys that the prevalence of insomnia among college students increase significantly during COVID-19, and poor sleep is the most prominent sleep problem among college students in China [64]. College students are reported to have poor sleep quality, including difficulty falling asleep, waking up early, lack of sleep, and even frequent nightmares and drowsiness [65], which significantly reduces the mental health of college students [66]. In traditional Chinese culture, sleep is regarded as an important means to maintain physical and mental health. According to the ancient Chinese book Huangdi Neijing (Yellow Emperor’s Inner Classic), regulating work and rest according to natural law can make people’s mental state healthier. The ancient Chinese people improved sleep quality by regulating diet, exercise, psychological regulation, environmental regulation, herbal regulation and other ways. College students should develop healthy sleep habits, change the habit of staying up late and getting up late, stop playing with mobile phones before going to bed and arrange sleep time reasonably. Confucius, the founder of Confucianism in China, emphasized the importance of sleep many times in the Analects of Confucius. Only by ensuring adequate sleep, can we have a healthier body and mind. College students can improve their sleep quality by creating a comfortable sleep environment, ensuring a regular sleep time of 7–8 h per day, and appropriate exercise to promote sleep quality.

The study also found that good interpersonal relationship significantly reduced depression of college students, with an average reduction of 2.1%. The results are consistent with other studies [67]. “Interpersonal relationship” is a compulsory course in college. Studies have shown that forming and maintaining stable interpersonal relationships is a basic human motivation [68]. After entering the colleges, college students begin to contact with more diverse and different groups of people. College students should enhance their interpersonal skills, so as to make their study and life more enjoyable and better withstand the psychological impact of adverse events. Research results consistently show that poor interpersonal relationships are associated with negative mental health in young people [69, 70]. Higher levels of intimacy in peer relationships are associated with healthier psychological outcomes [71], while negative friendship quality increases depressive symptoms [72]. The harmonious relationship between people is also the most important aspect of Confucianism. Confucius took benevolence as the core of his thought, which played an important role in building the harmonious interpersonal relationship of contemporary people. Chinese traditional culture tells us that when dealing with interpersonal relationships, individuals should do a good job of their own moral cultivation, understand things with harmonious thinking, and treat people with harmonious attitudes. College students can maintain a good interpersonal relationship from the following aspects: firstly, they should respect others and be ready to help others. Secondly, they can attend the students’ party appropriately to enhance the relationship and make regular plans to meet and interact with others to build strong relationships. Finally, they should have a grateful heart and praise others sincerely.

In conclusion, under the special environment of the COVID-19 pandemic, it is necessary to mobilize multiple entities such as the government, colleges and students to participate together to improve the mental health of college students.

### Limitations and future research

This study also has some limitations. Data in this study were collected during the period of strict control of the pandemic in China. The use of maturity scale and large sample data is representative, which helps us to understand the influence of academic stress on depression of Chinese college students in a specific period. However, the study has the following limitations. This is a cross-sectional study. Due to data limitations, the impact of academic stress on the depression of college students cannot be seen in the long term. There may also be a problem of selection bias, that is, there may be a problem of non-randomness of the sample, which may bring about an overestimation (or underestimation) of the results. In addition, this study uses questions instead of questionnaires to evaluate some variables, which is reasonable, but there may be some bias. Therefore, future research needs to further explore prospective longitudinal studies, conduct long-term follow-up investigations, conduct larger surveys as far as possible, and consider using more scales for further verification and analysis.

## Conclusion

Using the data of Chinese college students during the COVID-19 pandemic, we found a causal relationship between academic stress and depression. The influence of academic stress on the depression of college students had a difference in work experience as student cadres, which showed that college students who had served as student cadres were less affected by academic stress, college students who had not served as student cadres were greatly affected by the academic stress. In addition, college students’ attitudes toward COVID-19 and counselors’ concerns for the students had significant effects on college students’ depression. Positive coping style, high quality sleep and good interpersonal relationship were the important mechanisms of the impact of academic stress on college students’ depression. This is very important for us to develop targeted interventions. It is hoped that our study will stimulate further exploration of how academic stress affects mental health in specific periods in the future.

## Data Availability

The data and materials can be obtained from the corresponding author.
